# Human papillomavirus oncogenic expression in the dysplastic portio; an investigation of biopsies from 190 cervical cones

**DOI:** 10.1038/sj.bjc.6601691

**Published:** 2004-03-02

**Authors:** I Kraus, T Molden, L E Ernø, H Skomedal, F Karlsen, B Hagmar

**Affiliations:** 1 Institute of Pathology, The National University Hospital, 0027 Oslo, Norway; 2 NorChip, 3490 Klokkarstua, Norway; 3 Department of Gynaecology and Obstetrics, Østfold County Hospital, 1603 Fredrikstad, Norway

**Keywords:** human papillomavirus (HPV), oncogene, gene expression, NASBA, PCR, cervical intraepithelial neoplasia (CIN)

## Abstract

In this study, we investigated the presence of E6/E7 transcripts of seven common high-risk human papillomavirus (HPV) types in 190 cervical biopsies. The RNA-based real-time nucleic acid sequence-based amplification assay (NASBA) and type-specific PCR, both detecting HPV 16, 18, 31, 33, 45, 52, and 58, as well as consensus PCR, were performed on all 190 biopsies. High accordance between type-specific and consensus PCR confirms that the HPV types included in this study are the most common types present in cervical dysplasia. Furthermore, we see a clear increase in the incidence of HPV, both DNA and RNA, along with the histological severity of dysplasia. HPV RNA was detected in all but two PCR-positive cases, confirming that the virus exerts E6/E7 mRNA expression in cases of high-grade dysplasia. Out of 19 women given a normal or borderline diagnosis at conisation, only four were found HPV positive, which may suggest that unnecessary conisations can possibly be reduced by introducing HPV testing into the preoperative routine assessment.

Human papillomavirus (HPV) infections are associated with the development of cervical neoplasia and are a necessary factor in the aetiology of cervical carcinoma (Walboomers *et al*, 1999; Bosch *et al*, 2002; zur Hausen, 2002). The most frequent HPV types found in high-grade cervical intraepithelial dysplasia (CIN II/III) and in cervical carcinomas are HPV 16, 18, 31, 33, and 45, which are often referred to as high-risk HPV or cancer-associated HPV types. However, the association between a positive HPV DNA test and the risk of subsequent development of dysplasia is partly unknown; the presence of HPV DNA does not necessarily indicate a risk for developing a more severe lesion. In fact, HPV is a very common virus among sexually active women and most infections are transient and asymptomatic (zur Hausen, 1991; Ho *et al*, 1998; Moscicki *et al*, 1998). Only very few infections cause morphologic changes in the epithelium and only a small percent of these will eventually develop cervical cancer (Bosch *et al*, 1995). On the other hand, several studies have shown that in cervical carcinogenesis, the expression of HPV E6 and E7 ORF is required for cell transformation and immortalisation (zur Hausen, 1988; Munger *et al*, 1989; Stoler *et al*, 1992). Consequently, persistent expression of these oncogenes may serve as an indicator of progression to CIN and invasive cancer (Sotlar *et al*, 1998), and detection of E6 and E7 mRNA transcripts may therefore be of higher prognostic value than the detection of HPV DNA. Moreover, new methods for preservation of RNA in cells have made gene expression analysis more feasible.

In this study, we investigated the presence of high-risk HPV E6/E7 transcripts in cervical biopsies from patients undergoing conisation. The analysis was performed on a biopsy taken from the ectocervix/transformation zone at the time of conisation, and the pathological diagnosis of this particular biopsy was not always representative for the existing lesion that was later revealed in the cone. This strategy gave us access to a uniformly preserved material with varying degrees of squamous cell dysplasia, yet from cones that in most cases harboured a CIN III lesion. For RNA detection, the RNA-based real-time nucleic acid sequence-based amplification assay (NASBA) was used, focusing on the reportedly most common oncogenic HPV types 16, 18, 31, 33, 45, 52, and 58 (Walboomers *et al*, 1999; Munoz *et al*, 2003). Results were compared with cytologic and histopathologic findings, and HPV DNA status as assessed by consensus and type-specific PCR.

## MATERIALS AND METHODS

### Cell culture

The cervical carcinoma cell lines SiHa (squamous cell carcinoma), CaSki (squamous cell carcinoma), and HeLa (adenocarcinoma), used as positive controls, were obtained from the American Type Culture Collection, USA. The SiHa and HeLa cell lines were maintained in Dulbecco's modified Eagle's medium (DMEM), CaSki cells in RPMI medium, supplemented with 10% fetal bovine serum (FBS), 2 mM L-glutamine, and 25 *μ*g ml^−1^ gentamicin. The cells were incubated at 37°C in a 5% CO_2_ atmosphere. The cells were harvested directly in lysis buffer (bioMérieux, the Netherlands, containing 5 M guanidine thiocyanate).

### Patients/clinical samples

The study was performed on 190 consecutive biopsies, taken from 190 women admitted to Østfold County Hospital for treatment of CIN during the period October 1999 to October 2001. The mean age at diagnosis was 37.4 years (range 22–74 years). Biopsies were frozen at −70°C immediately after collection. All women were subjected to subsequent conisation.

### Cytological and histological examination of samples

Initially, routine cytological grading was performed according to local routine guidelines. No review cytology was performed. After a high-grade cytology report, or an equivocal cytological smear, the patients were referred to colposcopy and biopsy (Østfold County Hospital). Iodine painting of the portio was used as a means of visualising dysplasia. Multiple biopsies were taken from the external os and from any colposcopically pathological lesion. In addition, the cervical canal was scraped. If these biopsies (Biopsy 1/Histology 1, Table 1

**Table 1:** Cytology and histology results

	**Cytology**	**Histology 1 (Biopsy 1)**	**Histology 2a**	**Histology 2b (Biopsy 2)**	**Histology 3**
Squamous cell carcinoma		2			
Adenocarcinoma		1			
CIN III	152	151	53	37	158
CIN II	28	17	30	30	13
CIN I	3	6	32	14	6
HPV/condyloma/koilocytosis			4	7	
Uncertain benign/malignant^a^	3				1
Benign with control^b^	2	11			2
Normal			71	11	7
Inadequate		2		3	3
Not representative for transformation zone				88	
Uncertain diagnosis	2				
					
Total	190	190	190	190	190

a Corresponding to Bethesda nomenclature ASC-H.

b Corresponding to Bethesda nomenclature ASC-US.

) confirmed a high-grade lesion (CIN II/III), the patient was again admitted to hospital, this time for conisation. In some cases, the patients were referred to conisation on the basis of an unclear diagnosis or a long-lasting persistent CIN I lesion. For these cases, only a diagnostic cone was taken, as small as possible. If the woman did not plan for future pregnancy, a combined diagnostic and therapeutic conisation was performed. Before conisation, but after local anaesthesia was applied, a single biopsy (Biopsy 2/Histology 2b, Table 1) was taken from the ectocervix. This biopsy (2 × 2 mm) was frozen within 2 min at −70°C. Biopsy 2 was split in two when frozen, and half was used for DNA/RNA extraction. The other half was fixed in 10% buffered formaldehyde and processed for histopathological examination. Some lesions were not correctly oriented in the paraffin block and had to be reoriented or serial sectioned in order to show the relevant surface epithelium. Consequently, it cannot be guaranteed that the histopathological diagnosis reflect the actual condition of the tissue used for extraction. Finally, the cone specimen was evaluated by a histopathologist, who subsequently confirmed or disproved the presence and severity of dysplasia (Histology 3, Table 1).

### Extraction of nucleic acids

Nucleic acids were isolated using the automated NucliSens Extractor (bioMérieux, the Netherlands) as previously described (Boom *et al*, 1990). Briefly, the material was divided into smaller pieces while kept on dry ice (−80°C) and subsequently transferred to 1 ml of lysis buffer (bioMérieux) followed by 20 s of homogenisation using disposable pestles, after which 100 *μ*l of the sample was further diluted 10-fold in lysis buffer. A 100 *μ*l volume was then used for the DNA/RNA extraction, which resulted in a final elution volume of −40 *μ*l. Extracts were stored at −70°C.

### NASBA protocol: amplification and detection of HPV RNA

Human papillomavirus RNA was detected by real-time NASBA (Compton, 1991; Chan and Fox, 1999; Deiman *et al*, 2002). Reagents were obtained from NorChip (Klokkarstua, Norway), as they are presented in the commercially available PreTect HPV-Proofer kit. Briefly, NASBA is based on isothermal RNA amplification, accomplished by the simultaneous enzymatic activity of avian myeloblastosis virus (AMV) reverse transcriptase, T7 RNA polymerase, and RNase H (Compton, 1991). For detection we used primers and molecular beacon (MB) probes directed against E6/E7 mRNA for HPV types 16, 18, 31, 33, 45, 52, and 58 (NorChip, Klokkarstua, Norway). Final concentration of MBs used in the reaction was 2.5 mM. The NASBA amplification was carried out in a volume of 20 *μ*l at 41°C for 2 h. A 5 *μ*l volume of nucleic acids, diluted five times after extraction, was included in the reaction. As performance control, to avoid false negatives due to degradation of nucleic acid, we used a primerset and probe directed against the human U1 small nuclear ribonucleoprotein (snRNP)-specific A protein (U1A mRNA) (Compton, 1991; Nelissen *et al*, 1991). All samples were run in duplicate on separate microplate readers (Lambda FL-600, Bio-Tek Instruments, Winooski, USA). RNA isolated from CaSki/SiHa or HeLa cells served as positive controls for HPV 16 and HPV 18 transcripts, respectively. For the other HPV types, these cell lines were used as negative controls. In addition, negative controls consisting of all reagents except RNA were included for every seventh reaction.

### Human papillomavirus DNA analysis: polymerase chain reaction

The same nucleic acid extracts and amounts as used in the NASBA reaction were used for HPV DNA PCR. The L1 consensus primers Gp5+/Gp6+ (Roda Husman *et al*, 1995) were used to assess the samples that contained HPV DNA. The PCR amplification was carried out in a volume of 50 *μ*l containing as final concentrations 75 mM Tris-HCl (pH 8.8), 0.01% Tween, 20 mM (NH_4_)_2_SO_4_, 1.5 mM MgCl_2_, 0.2 mM dNTPs, 50 pmol of Gp5+ and Gp6+ primers, and 1.0 U of *Taq* DNA polymerase (Sigma-Aldrich, Missouri, USA). Human papillomavirus DNA was amplified by using a thermo cycler heat block from MWG, Ebersberg, Germany (Primus 96 HPL block). The first DNA denaturation was carried out for 2 min at 94°C, followed by 40 cycles of denaturation for 1 min at 94°C, annealing for 2 min at 40°C, and extension for 1.5 min at 72°C, prior to a final extension for 4 min at 72°C. Genotyping of HPV was performed by using PCR type-specific primers against HPV types 16, 18, 31, 33, 45, 52, and 58 (NorChip, Klokkarstua, Norway), utilising 25 pmol of each primer. The PCR conditions were 35 cycles of denaturation for 0.5 min at 94°C, annealing 0.5 min at 57°C, and extension for 1 min at 72°C. For a cellular control, we used primers against the human *β*-globin gene (Mwenda *et al*, 1999). DNA isolated from CaSki/SiHa or HeLa cells served as positive controls for HPV 16 and HPV 18, respectively. For the other HPV types, these cell lines were used as negative controls. Negative controls, consisting of all reagents except DNA, were included for every seventh reaction. Fragments were visualised by using the 2100 Bioanalyzer Multi-Instrument system (Agilent Technologies, CA, USA).

### DNA sequencing

Samples that tested HPV DNA positive with the consensus primers yet negative with type-specific PCR primers were sequenced in order to determine the HPV type. Sequencing was performed at the University of Oslo (Norway) by the MegaBACE Sequence Analyzer (Amersham Biosciences, Little Chalfont, England) according to in-house protocols. Sequence comparisons were performed using BLAST.

## RESULTS

### Cytology and histology

Table 1 summarises the clinical diagnoses of patients included in the study. According to cytology, 180 out of 190 patients had indications of high-grade dysplasia (CIN II or III); eight had repeatedly the diagnosis of mild or low-grade abnormalities, and two patients had an equivocal cytological smear. In all cases, the cytological report was the basis for subsequent hospital admittance, colposcopy and biopsy (Histology 1, Table 1). A high-grade lesion (CIN II or III) was in 168 out of 190 cases confirmed by histological examination. All patients having a high-grade lesion either at cytology or histology were admitted for conisation. In addition, some patients were admitted for conisation on the basis of equivocal cytology and histology. Biopsy 2, taken at conisation (Histology 2b, Table 1), was used for RNA analysis. Only one biopsy was taken, and because local anaesthesia made it difficult to locate the lesion by colposcopy, this biopsy was not always representative for an existing CIN lesion that was later revealed in the cone. This biopsy was examined twice by a pathologist who was blinded to the subject's HPV status. The first histological examination (Histology 2a, see Table 1) diagnosed only 83 samples of the original 168 as CIN II/III, 32 as CIN I, and four as HPV/condyloma. In 71 samples, high-grade lesions were not detected. Re-examination of the slide, to check for representativeness (Histology 2b), revealed 88 of the biopsies as not representative, that is, the tissue was not taken from the transformation zone; CIN III was found in 37 cases, CIN II in 30 cases, and CIN I in 14 cases. Conisation confirmed 158 of the women to harbour a CIN III lesion, 13 having CIN II. As many as 19 women had a low-grade or equivocal diagnosis at conisation (Table 1): six were diagnosed as CIN I, three as nonrepresentative, and 10 were diagnosed as benign or normal. Almost all of these women were given a high-grade diagnosis at cytology, but were given a low-grade/benign diagnosis at the first histological examination (Histology 1); four of these 19 women were found to be HPV positive (Table 2

**Table 2:** Samples diagnosed as normal or borderline at conisation (Histology 3), including previous cytological and histological diagnoses, as well as NASBA and PCR results

**Cytology**	**Histology 1 (Biopsy 1)**	**Histology 2a**	**Histology 2b (Biopsy 2)**	**Histology 3**	**NASBA**	**PCR**	**Gp5+6+**
CIN III	Benign with control	Normal	Nonrepresentative	Normal	–	–	–
CIN III	Benign with control	Normal	Normal	Normal	–	–	–
CIN III	Benign with control	CIN I	Nonrepresentative	Normal	–	–	–
CIN III	Benign with control	CIN I	Nonrepresentative	Normal	–	–	–
CIN III	Benign with control	CIN I	Nonrepresentative	Normal	31	31	+
CIN II	Benign with control	Normal	Nonrepresentative	Normal	–	–	–
CIN III	Benign with control	CIN I	Nonrepresentative	Normal	–	–	–
CIN III	Inadequate	CIN I	CIN I	Inadequate	–	–	–
Uncertain benign	Inadequate	Normal	Nonrepresentative	Inadequate	–	–	–
Uncertain benign	CIN III	Normal	Normal	Inadequate	–	–	–
CIN III	Benign with control	Normal	Nonrepresentative	Benign with control	–	–	–
CIN III	Benign with control	CIN I	HPV	Benign with control	–	–	+^a^
CIN II	Benign with control	Normal	Nonrepresentative	Uncertain benign	16	16	+
CIN II	CIN I	Normal	Nonrepresentative	CIN I	–	–	–
CIN I	CIN I	CIN II	Koilocytosis	CIN I	–	–	–
CIN III	CIN I	CIN I	Nonrepresentative	CIN I	–	–	–
CIN III	CIN I	CIN I	Nonrepresentative	CIN I	–	–	–
CIN III	CIN I	Normal	Normal	CIN I	16	16	+
CIN III	CIN I	Normal	Normal	CIN I	–	–	–

a By sequencing revealed as HPV 35;

–negative test result;

Non-representative=not representative for the transformation zone.

).

### Human papillomavirus RNA and DNA in CIN

**Figure 1:**
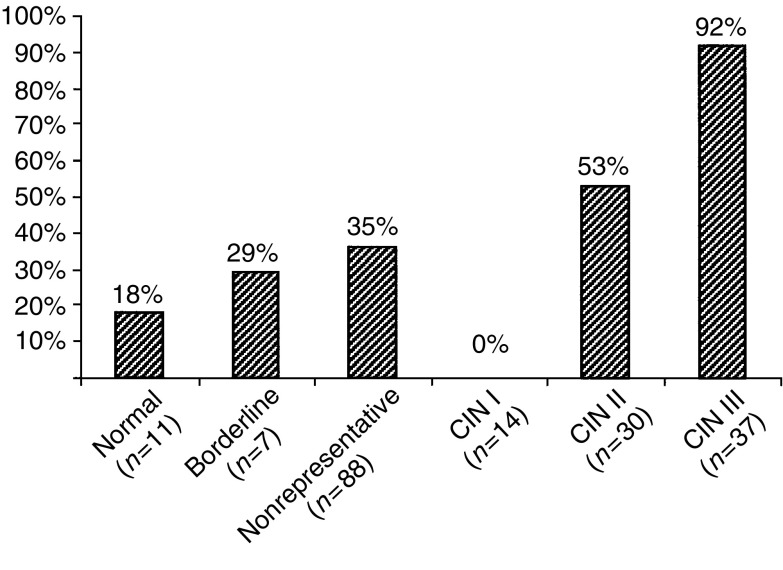
Oncogenic HPV activity detected by real-time NASBA. The presence of HPV DNA and RNA increased with the histological severity of the cervical intraepithelial neoplasia (CIN). HPV was also detected in samples with histological diagnosis normal, borderline, or nonrepresentative. The respective values in percentage are indicated above each bar. Borderline samples include HPV, condyloma, and koilocytosis.

Human papillomavirus E6/E7 transcripts from HPV 16, 18, 31, 33, 45, 52, and 58 were detected in 88 out of 190 (46%) biopsies, of which 83 out of 88 (94%) represented HPV 16, 18, 31, 33, and 45. Of the 37 samples diagnosed as CIN III (Histology 2b) 34 cases (92%) showed E6/E7 expression, only representing HPV 16, 18, 31, 33, and 45. In addition, we found oncogenic expression in 16 out of 30 cases (53%) with CIN II. No CIN I cases (*n*=14) were found to contain HPV RNA. Transcripts were also found in two out of seven cases of borderline samples, in two out of 11 cases not showing dysplasia, and in three samples being histologically inadequate. Among the 88 nonrepresentative samples, we detected HPV RNA in 31 samples (35%). Results are presented in Figure 1

. Figure 2

**Figure 2:**
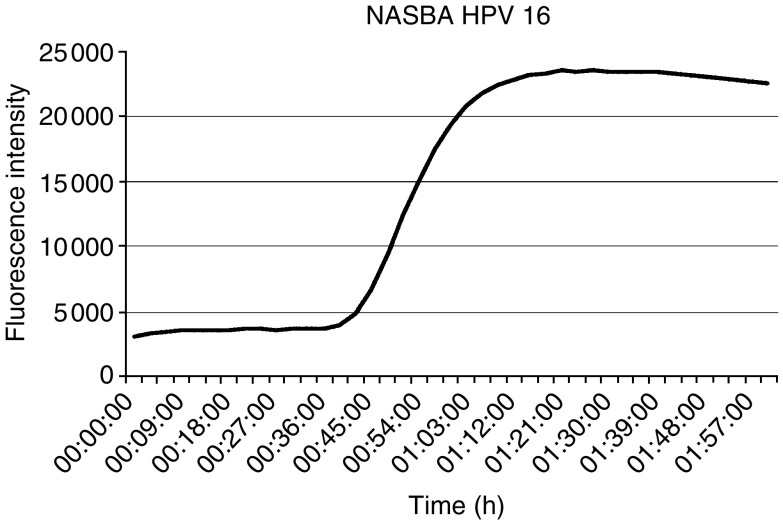
Amplification of HPV RNA by real-time NASBA. A positive reaction result is revealed as a sigmoid curve.

shows a typical RNA amplification curve as measured by real-time NASBA, exemplified by an HPV 16-positive sample.

In order to investigate the prevalence of HPV DNA *vs* RNA in the cervical biopsies, and to evaluate the analytical specificity and sensitivity of the NASBA method, we also performed PCR on the same 190 biopsies, both by type-specific and consensus PCR. Results are summarised in Table 3

**Table 3:** NASBA results shown according to PCR positives and negatives (both consensus and type-specific), for each of the histological categories

**Histology 2b**	**PCR positives (n=95)**		**PCR negatives (n=95)**	
	**No. of NASBA positives**	**No. of NASBA negatives**	**No. of NASBA positives**	**No. of NASBA negatives**
CIN III (*n*=37)	34	2^a^	0	1
CIN II (*n*=30)	16	1^b^	0	13
CIN I (*n*=14)	0	1^c^	0	13
Borderline^d^ (*n*=7)	2	1^e^	0	4
Normal (*n*=11)	2	0	0	9
Inadequate (*n*=3)	3	0	0	0
Nonrepresentative (*n*=88)	31	2^f^	0	55
				
Total (*n*=190)	88	7	0	95

Non-representative=not representative for the transformation zone.

a HPV16+HPV35.

b HPV67.

c HPV16.

d Borderline samples include HPV, condyloma, and koilocytosis.

e HPV35.

f HPV35+HPV51.

. Type-specific PCR, directed against E6/E7 for the HPV types 16, 18, 31, 33, 45, 52, and 58, detected 90 samples containing HPV, of which all but two were positive by NASBA. Using the consensus Gp5+/Gp6+ primers directed against the L1 gene, PCR detected HPV in 85 out of 190 biopsies, of which five were not detected by type-specific PCR. In addition, 10 samples that were positive by type-specific PCR were not detected by using consensus primers. In total, taking both consensus and type-specific PCR into account, PCR found 95 samples HPV positive.

The expression pattern for the different HPV types according to CIN grade is summarised in Table 4

**Table 4:** Distribution of different HPV types detected by NASBA

	**HPV types**						
	**HPV 16**	**HPV 18**	**HPV 31**	**HPV 33**	**HPV 45**	**HPV 52**	**HPV 58**
CIN III	25	3	4	3	2		
CIN II	8	1	2	4	1	1	1
Borderline	1						1
Normal	1						1
Inadequate	2					1	
Nonrepresentative	13	3	8	2	4	1	
							
Total (*n*=88)	50	7	14	9	7	3	3

Distribution of the seven high-risk HPV types determined by NASBA in the biopsies taken at conisation (Histology 2b). The total number is summarised to 93 due to multiple infections in five cases. Nonrepresentative=not representative for the transformation zone.

. Human papillomavirus 16 was the most common type found (50 out of 88), followed by HPV 31 (14 out of 88), HPV 33 (nine out of 88), HPV 18 (seven out of 88), HPV 45 (seven out of 88), HPV 52 (three out of 88), and HPV 58 (three out of 88). Five patients had multiple infections, all with a high-grade CIN diagnosis (CIN II or III): one patient had mixed expression of HPV 16 and HPV 18, one of HPV 16 and HPV 31, two of HPV 16 and HPV 33, and one of HPV 33 and HPV 58. HPV 52 and 58 were not detected in any CIN III cases. Table 5

**Table 5:** Distribution of high-risk HPV RNA for the age groups indicated

**Year of birth**	**No. of women (*n*=190)**	**HPV 16**	**HPV 18**	**HPV 31**	**HPV 33**	**HPV 45**	**HPV 52**	**HPV 58**
1926–1949	23	7	2	0	0	0	0	1
1950–1959	32	8	1	1	1	2	0	2
1960–1969	70	20	2	4	4	3	3	0
1970–1979	65	15	2	9	4	2	0	0

shows the distribution of HPV types for different age groups.

### DNA sequencing

The five samples positive by consensus PCR, yet negative by type-specific PCR and NASBA, were typed by sequencing. Three samples were revealed as HPV 35 (one CIN III, one borderline, one nonrepresentative), one sample as HPV 51 (nonrepresentative), and one sample as HPV 67 (CIN II).

## DISCUSSION

To our knowledge, this is the first study performing HPV mRNA analysis for several HPV types on a large number of histologically confirmed lesions. By comparing the RNA technology NASBA with conventional PCR of HPV DNA, this study permitted us to evaluate HPVs oncogenic activity in different grades of dysplasia. With a few exceptions, all patients included in the study were originally given a high-grade CIN diagnosis (CIN II/III) by cytological examination and/or by the first histological examination (Biopsy 1). However, the RNA analysis was performed on a separate biopsy (Biopsy 2), taken from the ectocervix/transformation zone at conisation. Only one biopsy was taken, and because local anaesthesia made it difficult to locate the lesion by colposcopy, this biopsy was not always representative for an existing CIN II/III lesion that was later revealed in the cone.

Biopsy 2 was examined twice histologically, in order to grade any dysplasia and to define samples not representative for the transformation zone, that is, samples containing only tissue from portio, external to the os (Histology 2a vs 2b, Table 1). The majority of the biopsies showing discrepancies in Histology 2a vs 2b presents small areas of metaplastic atypia, difficult to fit into the CIN groups (Geng *et al*, 1999; Montes *et al*, 1999; Ng *et al*, 2002; Zuna *et al*, 2002). The discrepancy in the relative frequency of ‘normals’ between Histology 2a and 2b is due to the fact that in 2b the emphasis was on representativeness for the transformation zone.

In the NASBA reaction, as well as in the type-specific PCR, we tested for the reportedly most common oncogenic HPV types 16, 18, 31, 33, 45, 52, and 58 (Walboomers *et al*, 1999; Munoz *et al*, 2003). We revealed that all but two PCR-positive cases also showed E6/E7 transcription. This shows that, at the level of severe dysplasia, HPV exerts an oncogenic activity (Nakagawa *et al*, 2000). Of the 37 samples diagnosed as CIN III (Histology 2b), we found 34 cases (92%) HPV RNA positive; three out of 37 CIN III cases did not show any oncogenic expression, one being PCR HPV 16 positive (Table 3). This sample may represent cell abnormalities prone to regression, as a consequence of HPV expression being switched off: regression of abnormal cytology in women with a positive HPV test at baseline is often associated with viral clearance (Nobbenhuis *et al*, 2001). This is, however, a field not widely explored and much remains to be learned about the mechanisms behind regression of cervical dysplasia. Furthermore, we see a clear increase in the incidence of HPV, both DNA and RNA, with the severity of the lesion (Figure 1), which is in accordance with previous studies (Sotlar *et al*, 1998; Nakagawa *et al*, 2000). The finding that no CIN I cases demonstrated HPV oncogenic expression suggests that, even in the presence of concominant CIN III in other areas, histologically defined CIN I may not be strictly related to HPV and precancerous disease (Crum *et al*, 1999). However, further studies are needed in order to confirm this assumption.

Human papillomavirus expression was not restricted to the CIN lesions. We also found oncogenic expression in two of 11 cases not showing dysplasia (Histology 2b, Table 1). In general, this may indicate a potential risk for the development of a high-grade lesion. In these cases, however, our finding most likely reflects the lesion confirmed by Histology 1, located in another part of the cervix. This indicates that the virus can be present in the cervix exerting an oncogenic activity before cell changes can be visualised histologically, supporting the view that the natural history of HPV includes periods before the manifestation of a cell abnormality (Moscicki *et al*, 1998; Nobbenhuis *et al*, 2001). Also among biopsies not representative for the transformation zone, as well as in inadequate samples, we detected a high rate of HPV DNA and RNA positivity (Figure 1).

In addition to type-specific PCR, we also tested the biopsies for HPV DNA using HPV consensus primers (Table 3). We made use of the primer set Gp5+/Gp6+, which detected all but 10 of the samples found positive by type-specific PCR. The ‘missed’ cases may be attributed to the fact that consensus primers are not sufficiently sensitive to detect all cases of HPV infection, at least not the cases containing a low HPV copy number (Karlsen *et al*, 1996). Besides, the use of these consensus primers may leave some important oncogenic HPV types undetected because of loss of the L1 sequence during viral integration. In clinical practice this can be critical, since integrated HPV 16/18 DNA has been revealed in various stages of dysplasia (Nagao *et al*, 2002; Gallo *et al*, 2003) and is shown to be common in patients with cervical carcinomas (Park *et al*, 1997; Kalantari *et al*, 1998; Walboomers *et al*, 1999). Therefore, the use of type-specific PCR, directed against one of the early-region genes (preferentially E6 and E7), is always recommended. On the other hand, consensus PCR detected five cases not detected by type-specific PCR. These samples were by sequencing revealed as HPV 35, 51, and 67. Nevertheless, the high association between consensus and type-specific PCR/NASBA supports that the HPV types included in this study are the most common types present in cervical dysplasia (Bosch *et al*, 2002).

The problem of inter- and intraobserver variability in cytology and histopathology paves the way for additional objective strategies in detecting cervical cancer precursors. Moreover, current management protocols may in certain cases lead to overtreatment (Meijer *et al*, 1998). This problem is emphasised in the present study: at the final evaluation of the cone, as many as 19 of the women received either a normal, benign, or low-grade diagnosis at (Histologi 3, Table 2). Almost all of these women had a high-grade diagnosis at cytology, yet a low-grade diagnosis at the first histological examination (Table 2). Conisation of these women was performed on the basis of high-grade cytological diagnoses, assuming the histology as being nonrepresentative. Only four of these women were found HPV positive, although we have to take into account that 12 out of 19 biopsies on which the HPV analysis were performed, were found to be nonrepresentative for the transformation zone. Nevertheless, unnecessary conisations can possibly be reduced by introducing HPV testing into the routine assessment. This is especially important among young women, who plan for future pregnancy, and for whom conisation entails increased risk for premature birth.

In conclusion, this study shows that HPV DNA and RNA detection achieve a similar result in biopsies of severe dysplasia, strengthening the case that HPV E6/E7 gene expression is necessary for oncogenesis in this region (zur Hausen, 1989). In general, detection of HPV E6/E7 transcripts, in combination with HPV typing, may have significant potential in identifying women who may be at a heightened risk for developing cervical dysplasia. However, our study unfortunately does not lend itself to calculations of clinical sensitivity and specificity, because of patient material selection and the fact that a relatively low number of true cytological and histological negative cases were included in the study. Therefore, RNA-based strategies for HPV testing are currently being investigated both in a screening context and in longitudinal follow-up studies.
